# Nanoparticles Carrying NF-κB p65-Specific siRNA Alleviate Colitis in Mice by Attenuating NF-κB-Related Protein Expression and Pro-Inflammatory Cellular Mediator Secretion

**DOI:** 10.3390/pharmaceutics14020419

**Published:** 2022-02-15

**Authors:** Elena K. Müller, Nataniel Białas, Matthias Epple, Ingrid Hilger

**Affiliations:** 1 Department of Experimental Radiology, Institute of Diagnostic and Interventional Radiology, Jena University Hospital, Friedrich Schiller University Jena, Am Klinikum 1, 07740 Jena, Germany; elena-mueller@posteo.de; 2 Inorganic Chemistry and Center for Nanointegration Duisburg-Essen (CeNIDE), University of Duisburg-Essen, Universitaetsstr. 5-7, 45117 Essen, Germany; nataniel.bialas@uni-due.de

**Keywords:** NF-κB p65, gene silencing, DSS-induced colitis, inflammatory bowel disease, ulcerative colitis, calcium phosphate, silica, nanoparticles

## Abstract

Ulcerative colitis is a disease that causes inflammation and ulcers in the colon and which is typically recurrent, and NF-κB proteins are important players during disease progression. Here, we assess the impact of silica-coated calcium phosphate nanoparticles carrying encapsulated siRNA against NF-κB p65 on a murine model of colitis. To this end, nanoparticles were injected intravenously (2.0 mg siRNA/kg body weight) into mice after colitis induction with dextran sulfate sodium or healthy ones. The disease activity index, the histopathological impact on the colon, the protein expression of several NF-κB-associated players, and the mediator secretion (colon tissue, blood) were analyzed. We found that the nanoparticles effectively alleviated the clinical and histopathological features of colitis. They further suppressed the expression of NF-κB proteins (e.g., p65, p50, p52, p100, etc.) in the colon. They finally attenuated the local (colon) or systemic (blood) pro-inflammatory mediator secretion (e.g., TNF-α, IFN-β, MCP-1, interleukins, etc.) as well as the leucocyte load of the spleen and mesenteric lymph nodes. The nanoparticle biodistribution in diseased animals was seen to pinpoint organs containing lymphoid entities (appendix, intestine, lung, etc.). Taken together, the nanoparticle-related silencing of p65 NF-κB protein expression could well be used for the treatment of ulcerative colitis in the future.

## 1. Introduction

Ulcerative colitis (UC) is an inflammatory bowel disease (IBD) that causes inflammation and ulcers in the digestive tract, which is thought to be the result of a complex interaction of environmental, genetic, and immune-regulatory factors. Inflammatory bowel diseases in general are characterized by an infiltration of neutrophils into the lesion of the colon accompanied by epithelial cell necrosis and ulceration [1], and patients mostly present with severe abdominal pain, fecal blood, weight loss, and diarrhea [2]. As chronic disorders, such inflammatory irregularities of the gastrointestinal tract are typically recurrent [3]. Their incidence has significantly increased globally, and there are currently no effective treatments available [4]. From the perspective of the side effects of systemic therapies, poor targeting of orally administered topical drugs, and low adherence to prescription represent frequent therapeutic challenges, i.e., there is a great need for innovative medicines for the treatment of inflammatory bowel diseases. In previous studies, the DSS (dextran sulfate sodium)-induced murine model has often been used in experimental investigations related to ulcerative colitis since it presents patho-morphological changes similar to human UC [5]. 

Several studies suggest that NF-κB plays an important role in sustaining a stable state of immunity in the gut, and disruptions in NF-κB are related to development of inflammatory responses [6,7]. Therefore, many of the current drugs being used for IBD treatments influence NF-κB regulation either directly or indirectly (e.g., corticosteroids, 5-aminosalicylates, anti-TNF monoclonal antibodies, etc.). Nevertheless, a high number of patients do not respond to these treatments [8], though the corresponding reasons are still not completely understood.

The NF-ĸB signaling pathway is known to be a key player in inflammation-associated diseases [9]. In non-stimulated cells, NF-κB is sequestered in the cytoplasm through direct interaction with a member of the IκB family of inhibitor proteins. It is activated by a diverse range of stimuli (immunoreceptors, cytokines, growth factors, and other stress impulses) [10]. An initiating stimulus leads to the activation of the so-called IKK complex, leading to phosphorylation of IκBα (IκBp) and its subsequent poly-ubiquitination and degradation. The liberated NF-κB dimer subsequently translocates to the nucleus, where it binds specific DNA sequences (for details, see reviews, e.g., [11]). Among the different NF-κB dimer components (e.g., p50, p52, p65), those encompassing p65 (RelA) are the critical players in regulating inflammatory responses in general [12] and in particular the secretion of several pro-inflammatory cytokines by mononuclear cells of the mucosal lamina propria [13]. In contrast to its pro-inflammatory role in myeloid cells, NF-κB has a protective role in intestinal epithelial cells, where it is required for maintaining epithelial integrity and intestinal immune homeostasis [14].

However, the suppression of p65 has remained a challenge so far, due to its cytoplasmatic localization. Therefore, the utilization of small interfering ribonucleic acid (siRNA) has been suggested to specifically degrade p65 mRNA after transcription by RNA interference (RNAi) and prevent its activity as a transcription factor thereafter. Since naked siRNA induces innate immune responses when applied intravenously in vivo [15], its encapsulation into nanoparticles has been suggested [16,17,18]. 

Particularly due to their unique and complex chemical, physical, and structural properties, nanoparticles (NPs) have turned out to be promising tools for the delivery of drugs to the body [19]. For siRNA delivery, so-called soft/organic NPs have been studied, whereby a direct ligand conjugation of small molecules, carbohydrates, aptamers, peptides, or antibodies with siRNA has been pursued to improve cellular uptake and target specific cell types. Additionally, liposomes and polymers have been developed as siRNA delivery carriers [20]. siRNA delivery was also investigated through the utilization of hard/inorganic NPs, whereby a polymer surface coating served to manipulate their solubility. In order to further improve targeted siRNA delivery, multiple coatings have even been designed. Typical hard/inorganic NPs have been made up of metals, metal oxides, and carbon materials (e.g., fullerenes, nanotubes, fibers) [21].

In this study, we investigated the impact of silica-coated calcium phosphate NPs carrying p65 siRNA in alleviating inflammatory processes in vivo, such as colitis in mice. We selected an inorganic nanoparticle delivery system since it is mechanically stable and, as such, it protects the integrity of the siRNA very well. Moreover, calcium and phosphates are naturally occurring components. We have previously shown that the mentioned NPs are functionally effective in cultured macrophages in vitro [22], but inflammation processes are associated with a tight interplay of several cell types not only involving macrophages. To this end, we investigated how such NPs influence first the clinical and histopathological features of murine model of colitis, and second, the expression of relevant NF-κB-associated proteins, as well as cytokine secretion in colon tissue. In order to further elucidate the corresponding therapeutic effects of NPs in the colon, we took a closer look into the protein expression in leucocytes versus that of the whole colon. In order to unveil the NP interaction with other immunologically relevant organs, we analyzed the spleen and mesenteric lymph nodes adjacent to the colon. We also assessed the influence of colitis on the NP biodistribution and studied the off-effects of NPs in the colon of healthy animals in order to show their biocompatibility in whole living organisms. 

## 2. Materials and Methods

### 2.1. Reagents for Nanoparticle Synthesis

For synthesis and preparation of near-infrared (NIR)-labeled bioactive calcium phosphate NPs, we used the following chemicals: calcium lactate pentahydrate (Sigma-Aldrich, St. Louis, MO, USA), diammonium hydrogen phosphate (VWR, Radnor, PA, USA), branched polyethyleneimine (PEI; *M*_w_ 25 kDa; Sigma-Aldrich, St. Louis, MO, USA), Dy734–NHS–ester NIR fluorophore (Dyomics, Jena, Germany), tetraethoxysilane (TEOS; Sigma-Aldrich, St. Louis, MO, USA), ethanol (99%; Fisher Chemicals, Hampton, NH, USA), aqueous ammonia solution (30–33%; Carl Roth, Karlsruhe, Germany), D-(+)-trehalose dihydrate (VWR, Radnor, PA, USA), hydrochloric acid (37%; VWR, Radnor, PA, USA), Dulbecco’s phosphate-buffered saline (DPBS; Gibco™, Carlsbad, CA, USA), and dimethyl sulfoxide (DMSO; Carl Roth, Karlsruhe, Germany). The NPs were biologically active due to the loading with therapeutic siRNA. siRNA against NF-κB p65 was obtained from Santa Cruz Biotechnology (Dallas, TX, USA). siRNA (sc-29411; *M*_w_ 13.8 kDa) was a mixture of 4 target-specific siRNA duplexes with the following sequences (from 5′ → 3′): CCAUGGAGUUCCAGUACUUtt, UCAGCACCAUCAACUUUGAtt, CGAAGUGCGUACACAUUCUtt, and GGAUUCCUGUACACCUUGAtt. To ensure maximal microbiological purity of the samples, for synthesis and handling of the NPs, ultrapure sterile nuclease-free water (Invitrogen, Waltham, MA, USA) was used, as well as Biosphere^®^ plus disposable laboratory materials (Sarstedt, Germany) and heat-sterilized and depyrogenated (250 °C, 1 h) glassware. All syntheses were performed in a nuclease-free environment (RNase AWAY™; Carl Roth, Karlsruhe, Germany). 

### 2.2. Nanoparticle Preparation

**Figure 1:**
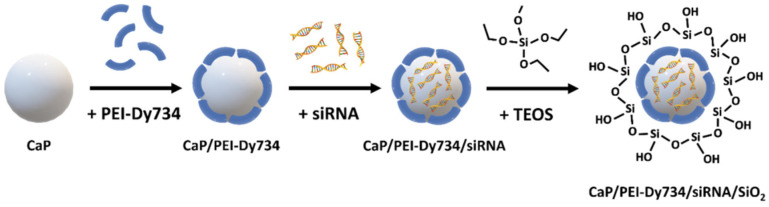
Schematic representation of the synthesis of Dy734-labeled and siRNA-loaded calcium phosphate nanoparticles (CaP/PEI-Dy734/siRNA/SiO_2_) for in vivo gene silencing of NF-κB p65 in mice. CaP—calcium phosphate, Dy—Dyomics, PEI—polyethyleneimine, siRNA—small interfering RNA, TEOS—tetraethoxysilane.

Fluorescent bioactive CaP NPs were prepared by wet chemical precipitation in a “bottom-up” approach, based on the protocols published earlier [22,23]. In brief, aqueous solutions of calcium lactate pentahydrate (18 mM; pH = 10), diammonium hydrogen phosphate (10.8 mM; pH = 10), and PEI–Dy734 (2 mg mL^−1^; diluted with unlabeled PEI in a volume ratio of one part PEI–Dy734 to four parts PEI) were simultaneously added with peristaltic pumps at a volume ratio of 5:5:7 mL min^−1^, respectively, to a strongly stirred beaker (100 mL) containing 20 mL water. The rapidly formed nanoparticle dispersion (CaP/PEI–Dy734) was stirred for the next 20 min at room temperature (RT) in darkness. For nanoparticle loading with siRNA, 0.9 mL of nanoparticle dispersion (CaP/PEI–Dy734) was mixed with 0.1 mL siRNA (0.36 mg mL^−1^) in a 1.5 mL reaction tube and stirred for 30 min at RT in darkness. Finally, to coat the NPs with a silica shell, 1 mL of fluorescent and biomolecule-loaded nanoparticle dispersion (CaP/PEI–Dy734/siRNA) was immediately added to a stirred mixture of 4 mL absolute ethanol, 5 μL TEOS, and 10 μL aqueous ammonia solution (7.8%) and further stirred overnight at RT under light exclusion. After this, the NPs (CaP/PEI–Dy734/siRNA/SiO_2_) were collected by centrifugation (4000 rpm; 30 min; RT), and the nanoparticle pellet was re-dispersed in 1 mL water, followed by vortexing and ultrasonication (cycle 0.8; amplitude 70%; 4 s). In the case of NPs without siRNA loading (CaP/PEI–Dy734/SiO_2_), the ultrasonication time was prolonged to 30 s. An amount of 0.04 mL D-(+)-trehalose dihydrate solution (0.5 g mL^−1^) was added to 1 mL of nanoparticle dispersion (CaP/PEI–Dy734/siRNA/SiO_2_) as cryoprotective agent, followed by shock freezing in liquid nitrogen and freeze-drying of the NPs for 72 h at 0.37 mbar and −10 °C. The NPs were stored at −80 °C until application. In order to obtain an adequate amount of fluorescent bioactive CaP NPs for in vivo gene silencing studies, we upscaled the above-described 1 mL synthesis by a factor of 20. All NPs were coated with a silica shell to protect the siRNA cargo (if present) and improve nanoparticle stability (Figure 1). For whole-body in vivo fluorescent imaging of nanoparticle-treated mice, the NPs were stabilized with Dy734-labeled PEI (see above). Unloaded fluorescent NPs (CaP/PEI–Dy734/SiO_2_) were prepared according to the bioactive NPs (skipping the siRNA loading step), as reference. 

### 2.3. Nanoparticle Characterization

Nanoparticle characterization was performed by using dynamic light scattering (DLS) and zeta potential (ζ) to determine nanoparticle size and colloidal stability (Zetasizer Nano ZS; Malvern Panalytical, Kassel, Germany). Scanning electron microscopy (SEM) was performed to study nanoparticle size and morphology (ESEM Quanta 400 FEG microscope; FEI, Hillsboro, OR, USA). Atomic absorption spectroscopy (AAS) was applied to determine the calcium content in the NPs (iCE 3000 M-Series spectrometer; Thermo Scientific, Waltham, MA, USA) after dissolution of the NPs in a 3:1 *v*:*v* mixture of H_2_O:HCl. The efficiency of nanoparticle loading with siRNA was determined by UV/Vis spectrophotometry (λ = 260/280; DS-11 FX+; DeNovix^®^, Wilmington, DE, USA). The endotoxin concentration in the NPs was assessed spectrophotometrically (Endosafe^®^ Nexgen-PTS™; Charles River, Wilmington, MA, USA). Fluorescence spectroscopy was used to confirm binding of the dye to the polymer and the fluorescence of NPs in the NIR wavelength region (λ_ex_ = 720 nm, λ_em_ = 749 nm) (Cary Eclipse; Agilent Technologies, Santa Clara, CA, USA). For DLS, ζ-potential determination, and fluorescence spectroscopy, aqueous dispersions of the NPs were used. SEM imaging was done on dried NPs. UV/Vis measurements were performed with supernatants obtained after nanoparticle centrifugation. For further data regarding instruments used for nanoparticle synthesis and handling, as well as detailed measurement parameters, see [22]. 

### 2.4. Animals, Animal Groups, and DSS-Mediated Induction of Colitis

**Figure 2:**
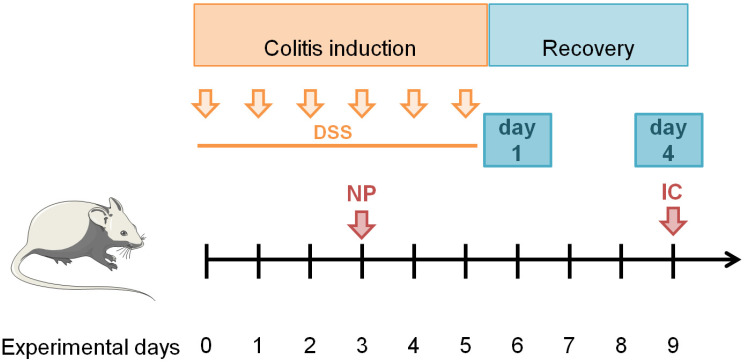
Timeline of in vivo experiments. NP—nanoparticles, IC—imaging of cathepsin activity in organs, DSS—dextran sulfate sodium (colitis inducer).

All animal experiments complied with the ARRIVE guidelines, were approved by the regional animal care committee, and were performed in accordance with state and federal guidelines on the ethical use of animals. Female BALB/cJRj mice (8 to 10 weeks of age) were purchased from Janvier Labs (CS 4105 Le Genest-Saint-Isle, 53941 CEDEX, Saint-Berthevin, France). For all necessary procedures, mice were sedated with 2% (*v*/*v*) isoflurane (Actavis Deutschland GmbH & Co., KG, Langenfeld, Germany). The induction of colitis in mice is generally induced by the oral administration of DSS (dextran sulfate sodium) via drinking water, which leads to inflammation in the mid-distal colon, decreased body weight, bloody diarrhea, and eventually death in a time- and dose-dependent manner [24]. During the induction phase, 3.75% (*w*/*v*) DSS (MP Biomedicals, Heidelberg, Germany) was added in the normal drinking water for 6 days (induction, experimental days 0–5). On experimental day 3, nanoparticle dispersions (2.0 mg siRNA/kg body weight in NaCl) were injected intravenously into the tail vein. The administered siRNA dose was selected according to a preliminary study; among the three doses tested (between 1.5 and 2.5 mg siRNA/kg body weight according to [25,26,27]), it was the one which best attenuated the development of colitis, as measured at experimental day 9 (Figure 2 and unpublished data). NPs were injected intravenously in order to target migrating phagocytes in the blood after their formation in the bone marrow in response to the DSS-induced colon inflammation. After induction of colitis, the inflammatory process was observed for another four days (recovery phase, experimental days 6–9). Animals were euthanized on recovery day 1 (experimental day 6) or 4 (experimental day 9) for further analysis of inflammation (Figure 2). 

### 2.5. Determination of Clinical Disease Parameters

The colitis severity during the experiments was assessed every day by scoring the disease activity index (DAI) according to [24,28] (Table 1). Blood samples (10 µL) were obtained at indicated days by punctation of the vena facialis. Diluted samples were used to measure a differential hemogram using a hematology analyzer (Sysmex Deutschland GmbH, Norderstedt, Germany). 

### 2.6. Determination of Pan-Cathespin Activity in Whole Organs via Ex Vivo NIRF Imaging

To visualize the inflammation process in relevant organs, the near-infrared (NIR) protease-sensing imaging agent ProSense680 (PerkinElmer LAS GmbH, Rodgau-Jügesheim, Germany) was used. This contrast agent detects the activity of extracellular cathepsins, whose dysregulation and overexpression are directly linked to inflammatory diseases. The agent is optically silent in intact state, but cleavage by cathepsin evokes high fluorescent activity [29]. ProSense680 (1.2–1.3 nmol per mouse) was injected into mice on experimental day 5 (last day of induction) intravenously into the tail vein. Imaging of inflammation was performed *postmortem* in relevant organs on experimental day 9 (recovery day 4) using an IVIS Spectrum CT (PerkinElmer LAS GmbH, Rodgau-Jügesheim, Germany) by acquiring a sequence of images with different combinations of excitation and emission filters (range: excitation, 640–745 nm; emission, 680–840 nm). Spectral unmixing of the ProSense680 spectrum and tissue autofluorescence was done according to the device’s manual. For semi-quantitative analysis, ROIs were placed in each organ or tissue (whole organ coverage) in order to extract the corresponding fluorescence intensities (as average radiant efficiency ([p s^−1^ cm^−2^ sr^−1^]/[µW cm^−2^])). 

### 2.7. Biodistribution of Nanoparticles

The biodistribution of the NPs was determined in diseased animals (see above) and compared to non-diseased ones (two groups, *n* = 10 each). In order to determine the early nanoparticle body distribution, NPs were injected intravenously in diseased or healthy animals on experimental day 3 (3 h post injection, where nanoparticle fluorescence is still detectable, concentration: 2.6 mg Ca^2+^/kg body weight, without siRNA). This dosage corresponds to the calcium concentration of administered siRNA-NPs with a dose of 2.0 mg siRNA/kg body weight. Three hours afterwards, mice were sacrificed, and defined organs were resected and analyzed for their Dy734 fluorescence intensity (IVIS Spectrum) with spectral unmixing of the NP spectrum and tissue autofluorescence, with ROI settings as described above. The change in organ fluorescence intensity from diseased animals was calculated with respect to healthy ones (set to zero). 

### 2.8. Protein Expression in Tissue Samples

Proteins were extracted from colon tissue using RIPA buffer and mechanical disruption (gentleMACS; Miltenyi Biotec B.V. & Co., KG, Bergisch Gladbach, Germany). Colon and spleen tissue were dissociated using Dissociation Kits (both Miltenyi Biotec B.V. & Co., KG). Leucocytes were subsequently isolated using CD45 MicroBeads (Miltenyi Biotec B.V. & Co., KG). Isolated leucocytes were lysed with RIPA buffer for subsequent analysis of protein expression. The total protein concentration in lysates was measured using the Bradford assay. To facilitate the simultaneous detection of six different proteins, multi-strip Western blotting was performed [30]. Proteins were separated in multiple 10% (*w*/*v*) SDS-PAGEs (10–20 µg total protein per lane). Proteins were transferred to an Immobilon-P membrane (Merck Millipore, Darmstadt, Germany). Blocking of the membrane was performed using PurelockTM Blocking Buffer (VILBER LOURMAT Deutschland GmbH, Eberhardzell, Germany) for 1 h at RT. Membranes were then probed with the corresponding primary antibodies targeting p65 and Bcl-2 (both Cell Signaling Technology, Frankfurt am Main, Germany), IκB, iNOS, or COX-2 (all abcam, Cambridge, GB) (all 1:1000). After washing, membranes were incubated with the respective HRP-coupled secondary antibody mouse anti-rabbit (Dianova GmbH, Hamburg, Germany) or HRP-conjugated β-actin antibody (abcam, 1:7500–10,000; detection of loading control). To quantify the expression of the proteins in the different treatment groups, a densitometric analysis of Western blot bands was performed with the software Bio1D (Vilber Lourmat Deutschland GmbH, Eberhardzell, Germany). Each data set was first normalized to the housekeeping protein β-actin and then to group H (no NPs, healthy). 

### 2.9. Cytokine Secretion in Tissue and Blood Samples

To determine the amount of pro-inflammatory mediators in different samples, the LEGENDplex^TM^ Mouse Inflammation Panel (13-plex) (BioLegend, San Diego, CA, USA) was used according to the kit’s protocol. Whole colon protein lysates were used to determine total concentrations of cytokines immediately after dissection, as well as 24 h later (as described in [31]). Plasma samples acquired after euthanasia were used to measure systemic inflammation markers. For data analysis, the recommended LEGENDplex™ Data Analysis Software (Version 8, BioLegend, San Diego, CA, USA) was used. Concentration of measured cytokines for the colon and the ex vivo cultivated colon were normalized to the weight of the used colon piece for each sample. 

### 2.10. Histology

Tissue specimens were fixed in methanol-stabilized 5% (*v*/*v*) formaldehyde solution (Otto Fischer GmbH, Waldkirch, Germany), embedded in paraffin, and sliced into three-micron-thick sections. Tissue sections were either stained with hematoxylin and eosin (HE) or used for immunohistochemistry. Colitis scoring on HE-stained slices was performed by utilization of a four-category system in relation to the extent of inflammation and crypt damage in the intestinal wall, as well as the extent of crypt damage in the whole tissue section. To assess the presence of specific proteins, tissue slices were treated for antigen retrieval, blocked (Biotin Blocking System; BioLegend, Dedham, MA, USA), incubated with primary antibodies (anti p65 NF-κB, 1:1000; anti CD11c, 1:250; anti CD25, 1:50; anti CD3, 1:100; anti F4/80, 1:400, all from rabbit; Cell Signaling, Danvers, MA, USA), rinsed in Tris-buffered saline added with 0.1% (*w*/*v*) Tween 20 (TBST), and then incubated with a secondary antibody (anti-rabbit IgG from goat, 1:2250, Dianova, Hamburg, Germany). Streptavidin alkaline phosphatase (Southern Biotech, Birmingham, AL, USA) and a chromogen (Liquid Permanent Red; Dako, Glostrup, Germany) were used for antigen detection. Sections were counterstained with hematoxylin (Sigma-Aldrich, Karlsruhe, Germany). The presence of the respective proteins in blinded tissue slices from 3 animals per group was estimated semi-quantitatively by three different investigators by utilization of a four-category scoring system. 

### 2.11. Statistics

Statistical analysis was performed using SPSS Statistics (IBM Corporation, Armonk, NY, USA). All data were considered normally distributed, based on literature reports on normality distribution of the same values. An ANOVA and Dunnett’s t (2-sided) post hoc test was used to compare the experimental groups. Differences with *p*-values below 0.05 were considered as statistically significant. 

## 3. Results

**Figure 3:**
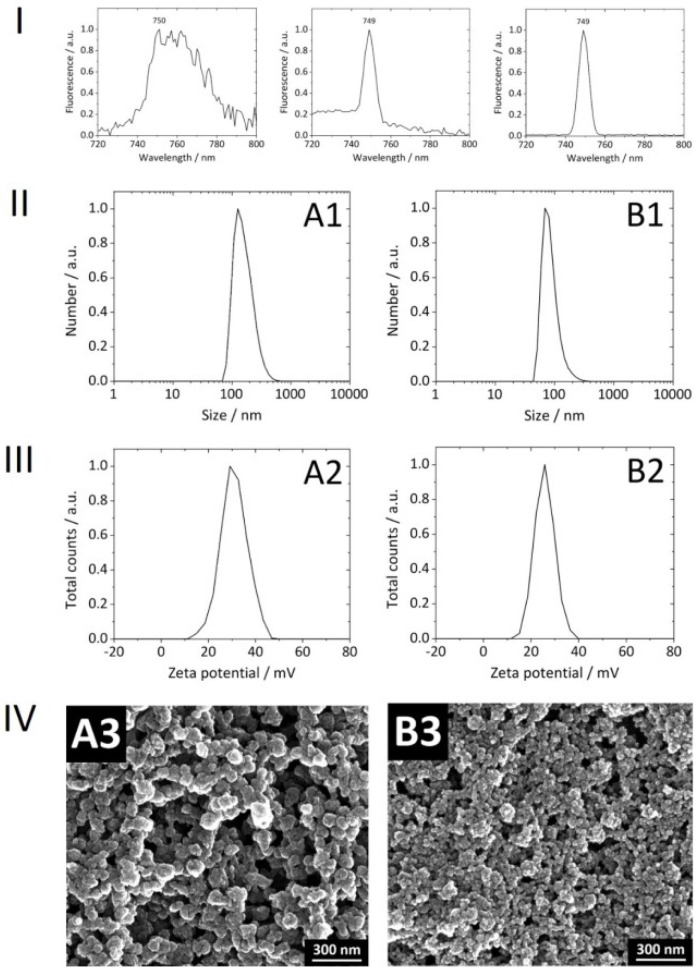
Physicochemical features of fluorophores and nanoparticles. (**I**) NIR fluorescence emission spectra of the free Dy734 dye (**left**), PEI–Dy734 complex (**center**), and representative reference Dy734-labeled calcium phosphate nanoparticles (CaP/PEI-Dy734/SiO_2_) (**right**) with marked emission maxima. The samples were excited at λ = 720 nm. The nanoparticle sample was diluted with water (1:4 *v*/*v*) for the measurement. (**II**) Normalized particle size distributions (**A1**). (**III**) normalized particle zeta potentials (**A2**). (**IV**) SEM micrographs of the representative nanoparticles (**A3**). The nanoparticle formulations are presented as follows: CaP/PEI–Dy734/siRNA/SiO_2_ (**A**) and CaP/PEI–Dy734/SiO_2_ (**B**). For detailed nanoparticle characterization, see Table 2.

With consideration of our NPs *per se*, the covalent attachment of Dy734 to PEI was successful. No loss of PEI during the conjugation reaction and subsequent purification was observed. The efficiency of Dy734–NHS–ester binding to PEI was approximately 80%. After coupling to PEI, there was no significant shift in the NPs’ NIR fluorescence emission maximum compared to the free dye, indicating no alteration in dye fluorescence after coupling to the polymer (Figure 3). The NPs were spherical, rather monodisperse, colloidally stable, and non-pyrogenic. Reference NPs without siRNA loading were smaller than the siRNA-loaded NPs (Table 2, Figure 3). The upscaling of the synthesis did not affect the physicochemical features of the NPs compared to the previous 1 mL synthesis [22]. The encapsulation efficiency of siRNA into the NPs was ∼95–100%. There was no loss of siRNA during purification via multiple centrifugation and re-dispersion steps. Lyophilized Dy734-labeled NPs, after re-suspension in water, showed a strong fluorescence signal (even when diluted) with a narrow dye-specific peak in the NIR region (Figure 3).

**Figure 4:**
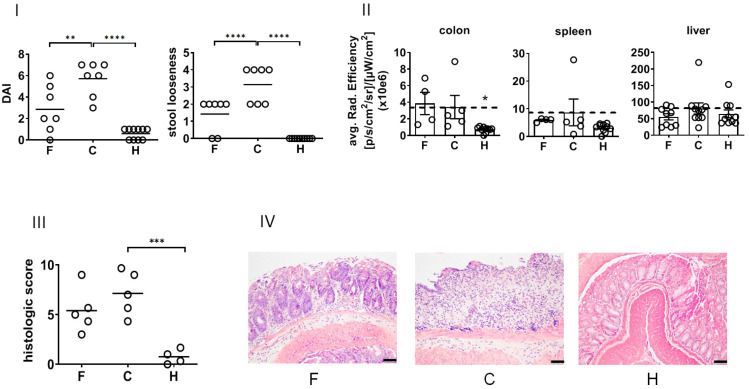
Impact of NPs on clinical and histopathological features of the DSS model of colitis in mice at recovery day 4. (**I**) Clinical features. (**II**) Ex vivo macroscopic organ imaging of pan-cathepsin activity as determined via near-infrared optical imaging after excision from animals (for details, see Methods). (**III**) Histopathological scoring of damages in the mucosa and submucosa (see Methods for scoring procedure) on the basis of HE-stained histological slides from the colon. (**IV**) Representative microscopic pictures of the colon (HE staining, scale bars: 50 µm). F—NPs in animals with colitis, C—colitis only (no NPs), H—healthy animals (no NPs). Mean ± SD; *n* = 5 to 7. Significant difference compared to group C with *p* < 0.05 (*), *p* < 0.01 (**), *p* < 0.001 (***), and *p* < 0.0001 (****).

In view of their biological effects in vivo, the treatment with NPs suppressed the DSS-induced DAI (recovery day 4, Figure 4I) and ameliorated stool looseness (in particular at recovery day 4, Figure 2) of DSS-induced colitis in mice. The NP treatment did not impacted the white blood cell count of diseased animals (Supplementary Figure S1). The macroscopic in vivo analysis of pan-cathepsin activity in whole organs revealed that NPs suppressed DSS-induced enzyme activity in the spleen and liver but not in the colon at the time of investigation (recovery day 4, Figure 4II). A closer analysis of the mucosa and submucosa of the colon showed that the treatment with NPs lowered the patho-morphological features of DSS-induced colitis (Supplementary Table S1, Figure 4III,IV).

**Figure 5:**
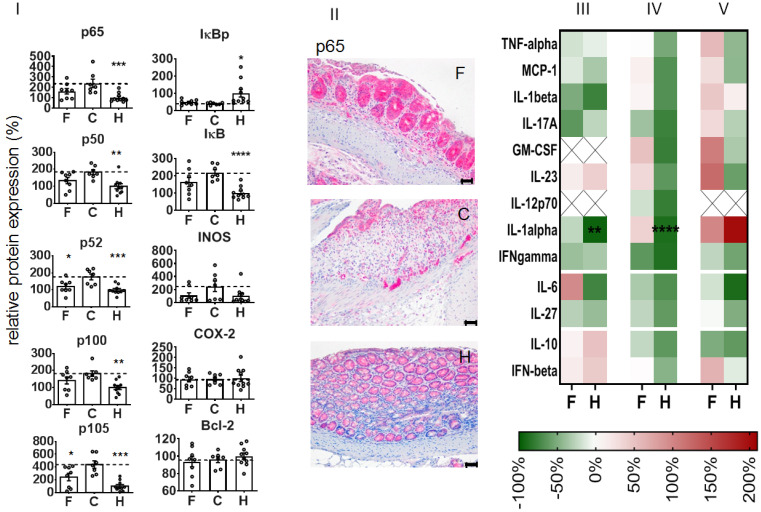
Impact of NPs on p65 NF-κB and related proteins in whole colon tissue during induction of DSS-mediated colitis in mice at recovery day 4. (**I**) Protein expression in whole colon tissue. (**II**) Representative microscopic pictures from immunohistochemical tissue staining showing the distribution of p65 (red, hematoxylin counterstaining: blue, scale bars: 50 µm). (**III**–**V**) Heat maps of cytokine expression in whole colon tissue (**III**), in ex vivo cultivated colon tissue, recovery day 5 (**IV**), and in blood plasma (**V**). Mediator secretion was related to levels in animals with colitis (in %, no NP administration); X—cytokine level below detection threshold. F—NPs in animals with colitis, C—colitis only (no NPs), H—healthy animals (no NPs). Mean ± standard error of the mean; *n* = 6 to 10. Significant difference compared to group C with *p* < 0.05 (*), *p* < 0.01 (**), *p* < 0.001 (***), and *p* < 0.0001 (****).

With regard to the expression of NF-κB-associated proteins, we observed that the administration of NPs attenuated the DSS-mediated increase of p65 and several other NF-κB players (e.g., transcription factor components p50 and p52, precursor proteins p100 and p105, regulatory kinase IκB) at recovery day 4. Additionally, iNOS protein expression was attenuated with regard to non-treated diseased animals, whereas there was no impact on the phosphorylated regulatory kinase IκB (IκBp), COX-2, or Bcl-2 expression (Figure 5I). Immunohistological studies showed that the expression of the latter proteins was particularly associated with the degree of DSS-induced morphological alterations in the colon, whereas infiltrated leucocytes (CD45+ cells) were predominately present in the mucosa (Supplementary Table S1). The treatment with NPs inhibited several pro-inflammatory cytokines (e.g., IL-1β, IL-17A, IFN-γ, IL-27, etc., related to non-treated animals with colitis, Figure 5III,IV) and transitorily stimulated the secretion of some other mediators (e.g., IL-6, GM-CSF, Figure 5III,IV) in the colon of DSS-induced colitis mice. The nanoparticle treatment further altered the mediator secretion in the blood plasma of diseased animals, with the exception of IL-10 and IFN-β (inhibition, Figure 5V), GM-CSF, and IL-23 (stimulation on recovery day 4, Figure 5V). 

**Figure 6:**
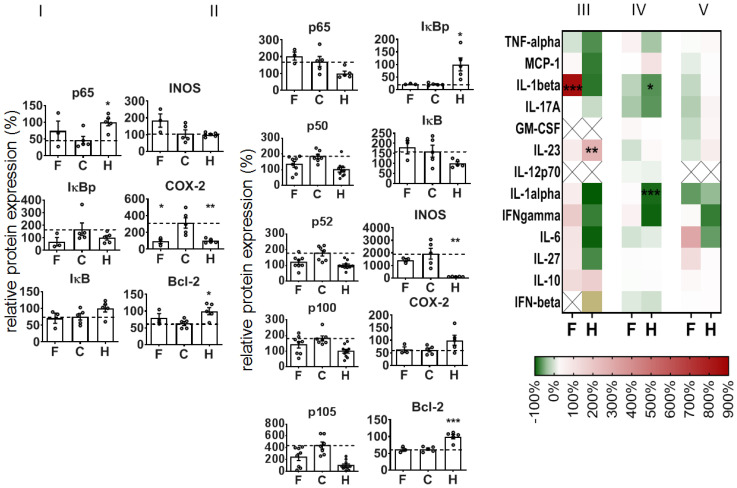
Impact of NP on p65 NF-κB and related proteins in the colon during induction of DSS-mediated colitis in mice at recovery day 1. Protein expression in leucocytes (CD45^+^ cells) (**I**) and in whole colon tissue (**II**); mean ± SEM; *n* = 3–5. (**III**–**V**) Heat map of cytokine expression in whole colon tissue (**III**), in ex vivo cultivated colon tissue (24 h, (**IV**)), and in blood plasma (**V**). Mediator secretion was related to levels in animals with colitis (in %, no NP administration); X—cytokine level below detection threshold. F—NPs in animals with colitis, C—colitis only (no NPs), H—healthy animals (no NPs). Mean ± SEM; *n* = 3 to 10. Significant difference compared to group C with *p* < 0.05 (*), *p* < 0.01 (**), and *p* < 0.001 (***).

To further understand the mode of action when administering NPs during colitis induction, we took a closer look at the colon on an earlier recovery day (recovery day 1), i.e., at a point in time close after internalization in cells and intra-cytoplasmatic delivery of siRNA [22]. Here, too, the treatment of mice with NPs was already attenuating the DSS-mediated DAI, the impact on the rectal bleeding, the colon length, and the histopathological features of colitis (Supplementary Figure S2). Curiously, we saw a different impact of the NPs on the expression of NF-κB-related proteins in colon leucocytes versus the whole colon tissue of animals with DSS-induced colitis (Figure 6I,II). With consideration of the colon leucocytes (CD 45^+^ cells), the NPs alleviated the DSS-mediated impact on p65, IκBp, and COX-2 protein expression, and they stimulated iNOS protein expression, but they had no distinct impact on DSS-mediated changes in IκB and Bcl-2 expression (recovery day 1, Figure 6I). In view of the whole colon tissue, the NPs had an alleviative impact on the DSS-induced increase in p50, p52, p100, and p105 (Figure 6II) protein expression. Furthermore, the NPs did not affect IκBp and IκB iNOS or Bcl-2 protein expression in diseased animals. With consideration of the cytokine and mediator expression, the administration of NPs only slightly increased the DSS-mediated mediator secretion of the colon (reddish in heat map Figure 6III,IV, e.g., IFN-γ), with the exception of IL-1β (red in heat map, Figure 6III) in whole colon and of IL-6 in blood plasma (Figure 6V). 

Our studies on the interaction of NPs with the spleen—as an immunologically relevant organ other than the ulcerated colon—showed that the NP treatment repressed the DSS-mediated increase in CD11c^+^ (dendritic cells), CD25^+^ (activated T cells), and CD3^+^ (total T cells) in this organ (Supplementary Figure S3II). A similar situation was encountered in relation to the adjacent mesenteric lymph nodes (Supplementary Figure S4). 

**Figure 7:**
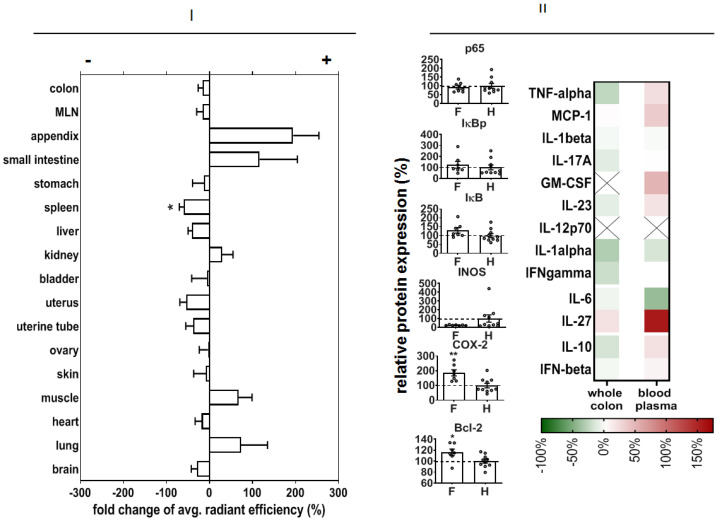
Off-target effects of p65 functional NPs after intravenous injection. (**I**) Changed accumulation of nanoparticles in colitis-bearing animals relative to healthy ones (fold changed NP accumulation, left). Nanoparticles were injected intravenously (2.0 mg siRNA/kg body weight in NaCl) at experimental day 3 in mice with DSS-induced colitis or in healthy mice. F—NPs in animals with colitis, H—healthy animals (with NPs). Mean ± SEM). (**II**) Impact on protein expression in the whole colon (left) and cytokine expression in colon tissue and blood plasma (right) on recovery day 4. Cytokine expression was related to the expression levels in non-treated healthy animals (in %, no NPs); X—cytokine level below the detection threshold. Mean ± SEM; *n* = 6 to 10. Significant difference compared to group H with *p* < 0.05 (*) and *p* < 0.01 (**).

In order to predict potential off-target effects after systemic administration, we analyzed the early organ distribution of NPs after intravenous injection in DSS-induced colitis in mice (experimental day 3, see Methods). There was a distinct NP accumulation, particularly in the small intestine and appendix, but also in muscle and lungs (Figure 7I). 

We also studied the off-effects of NPs in the colon of healthy animals in order to elucidate their biocompatibility in whole organisms. In this context, we observed that our NPs did not change p65, IκBp, or IκB protein expression, but they decreased iNOS protein expression and increased COX-2 and Bcl-2 protein expression (Figure 7II). Mediator secretion in whole colon tissue was not changed, but the level of IL-27 was increased with respect to non-treated healthy animals. 

## 4. Discussion

In our study, we found that NPs effectively alleviated the DSS-induced clinical and histopathological features of DSS-induced colitis. They further suppressed the DSS-induced increase of the several NF-κB-related proteins at recovery day 4. The alleviative effects of NPs on DSS-induced colitis in mice were detectable early on in colon leucocytes (recovery day 1). There was also an impact of NPs detectable in the spleen and mesenteric lymph nodes. Off-effects of siRNA in the colon of healthy animals were very low. 

With consideration of our CaP NPs, the siRNA was incorporated by surface adsorption (non-covalent attachment). The electrostatic attraction between siRNA and CaP NPs resulted from the complementary electrical charges of siRNA (negative) and NPs (positive due to PEI). In contrast, the silica shell was covalently attached to the nanoparticle surface by silanization (Stöber process). This is an efficient method to modify hydroxylated surfaces of materials and to enable their further superficial decoration (e.g., with ligands).

Regarding the size of our NPs, this parameter was studied by two independent methods: DLS and SEM. The size values determined by DLS were larger when compared to those obtained by SEM. This is due to the fact that in DLS, NPs are normally measured in a colloidal state (the hydrodynamic size is determined), whereas by SEM, the NPs are measured in a dried state (the size of the inorganic core without the hydration shell is determined). Furthermore, DLS is susceptible to nanoparticle aggregations, which is not the case in SEM. 

We expect that the siRNA encapsulated in our NPs was stable and remained bioactive during its intracellular processing, since (1) there was an efficient NF-κB p65 silencing effect after 72 h of incubation of the monocytes in vitro [22], (2) we used siRNA duplexes, which are much more resistant to the activity of nucleases than single-stranded nucleic acids, (3) the siRNA duplexes possessed 3′tt-ends (composed of deoxythymidine) that increased thermal stability of the siRNA and made it less susceptible to degradation by exonucleases, and (4) the siRNA encapsulation into NPs was stable (no loss of siRNA during synthesis and purification procedures).

Upon intravenous application, our NPs are expected to be captured by patrolling phagocytes of the blood, as well as those newly released from the bone marrow, as a result of the local inflammation caused by the microorganism infiltration in the colonic mucosa after its DSS-mediated destruction. Since the key pathways in the mobilization and recruitment of leucocytes are known to occur between the bone marrow and the regional lymph nodes, we expect that an important part of the injected NPs is located in the recruited phagocytes of lymphoid organs close to the colon.

According to our previous in vitro study [22], after uptake, the NPs are trapped in the endo-lysosomes of phagocytes and degraded, and the cargo siRNA is released to the cytoplasm, where it exerts its gene silencing action of p65 NF-κB transcription. 

Obviously, DSS-induced colitis per se affected cathepsin activity in the mentioned organs. Namely, extracellular matrix cathepsins are known to play an important role in inflammatory diseases since they are involved in matrix remodeling, in processing cytokines and chemokines, and in shedding several extracellular receptors and cell adhesion molecules during inflammation [32]. The fact that the treatment with NPs suppressed the DSS-induced cathepsin activity in the spleen and liver but not in the colon (recovery day 4) may well be explained by the extensive destruction of mucosal cells in the colon. At the same time, cathepsin activity was reduced in the liver and spleen as a consequence of mitigated systemic inflammatory processes after NP therapy. 

The NPs further suppressed the DSS-induced increase of p65 protein expression levels in whole colon tissue at recovery day 4. This decreased expression of p65 is well associated with a decreased transcription factor activity of p65 in the NF-κB-dependent signaling pathway. There was also an impact on other NF-κB transcription factor dimers, that is, the p50 and p52 protein expression, together with their precursors (p100 and p105) [33]. All mentioned changes pointed to the respective protein expression profile in healthy animals. Consequently, at recovery day 4, the NP-mediated inhibition of p65 (RelA) had an extensive impact on several important players of the NF-κB signaling pathway, particularly on the components of the transcription dimers involved in the canonical and non-canonical pathway; whereas the so-called canonical signaling pathway mediates a wide range of biological pathways, the non-canonical NF-kB pathway is involved in the cross-priming function of dendritic cells, as well as the generation and maintenance of T cells [34]. In the end, after NP treatment, there are fewer NF-κB dimers available for dimerization, translocation to the nucleus, and specific gene expression in the course of the inflammation processes, as a result of the DSS-induced mucosal destruction. 

The fact that the impact of the animal treatment with NPs on IκB and IκBp was less consistent (recovery day 4) might be the consequence of the fact that the mentioned proteins represent an important “regulation crossing point” in the course of the diverse NF-κB signaling pathways and that the inhibition of p65 may have led to biased activation/deactivation and degradation of IκB [35]. Additionally, the suppressed expression of iNOS after treatment of animals with DSS-induced colitis is an indication of reduced NO-mediated pro-inflammation potential in whole colon tissue, in particular since the inducible nitric oxide synthase (iNOS) is regulated by NF-κB activation [36]. Seeing as there was no impact on COX-2 protein expression, it is likely that our NPs had no distinct impact on prostaglandin production [37,38,39] at this recovery time point. At the same time, the low Bcl-2 protein expression indicates that cell death via apoptosis was not prominent in whole colon tissue. 

In agreement with their alleviative inflammatory potential in the DSS-mediated colitis mentioned above, the administration of the NPs inhibited several pro-inflammatory mediators. The local stimulation of IL-6 in the colon could well be related to repair processes in the mucosa, as it is known to stimulate intestinal epithelial proliferation and repair after injury [40]. The IL-6 secretion could be related, at least in part, to the presence of the NPs *per se*, since some studies postulate a nanoparticle-size-dependent effect on specific pro-inflammatory cytokine secretion (IL-1b, TNF-a and IL-6) [41]. Furthermore, the stimulation of GM-CSF secretion in the colon and blood plasma is a manifestation for macrophage maturation activities to promote anti-microbial functions in the colon [42]. The NP-mediated stimulation of the secretion of IL-23 is related to a promoted neutrophil recruitment after DSS treatment [43], and it is produced by dendritic cells and macrophages and fosters the regenerative activities of T helper 17 (Th17) and innate lymphoid cells [44]. Importantly, the treatment with NPs suppressed the secretion of IL-17A in whole colon tissue, which is associated with a protective role for the injured intestinal mucosa [45,46]. 

To further understand the mode of action when administering NPs during colitis induction, we took a closer look at the colon recovery day 1, when the siRNA was expected to be delivered intracellularly [22]. Here too, different leucocyte subsets were identified in the damaged colon mucosa (CD11c^+^, CD25^+^, and CD3^+^ cells), which reflects the presence of cellular immune responses for cleaning cellular debris and for initiating repair processes [47]. The presence of CD25^+^ cells is in agreement with their crucial role in inflammatory bowel pathogenesis [48]. Looking at the colon leucocytes (CD45^+^ cells), the early alleviation (recovery day 1) of DSS-induced changes in p65 protein expression via NP administration are ascribed to suppressed pro-inflammatory activities closely related to NF-κB activation pathways [34]. The distinct increase in iNOS expression in colon leucocytes is related to regenerative processes related to the production of NO to improve blood flow, as well as to reduce leucocyte and platelet recruitment and oxidative stress [49]. 

Beyond the mentioned observations in colon leucocytes on recovery day 1, the comparatively lower impact of NPs on the suppression of p65 in whole colon tissue is associated with biased activation processes as a consequence of the intense mucosal repair processes which were still ongoing. Namely, NF-κB signaling in whole colon tissue is required for tissue repair processes in general (i.e., initiation of reparative proliferation of mucosal epithelial cells, of extracellular matrix synthesis, etc.) [50]. It is not clear why the protein expression of several other players of the NF-κB signaling pathway (i.e., p50, p52, p100, and p105) was suppressed at the same time. Possibly, several interrelated and mutually exclusive mechanisms take place which regulate the stability and termination of nuclear NF-κB transcription factor dimers according to the specific physiological and/or metabolic requirements [12]. The suppression of the DSS-mediated increase in iNOS expression may well be related to oxidant stress as a result of mucosal injury, which does not have a distinct impact on their NF-κB activation as opposed to leucocytes (mononuclear cells [51]). The concomitantly stimulated secretion of several mediators in the whole colon tissue and the blood plasma was particularly related to IL-1β, IFN-γ, and IL-6, which reflect acute phase reactions and regeneration following acute colonic injury [52,53,54]. 

With consideration of the spleen, we show that the impact of NPs on spleen leucocytes is associated with a decreased presence of antigen-presenting and activated T cells. Being the second largest lymphoid organ, the spleen is involved in cellular immune response by controlling the level of blood cells (functionality and subtypes of neutrophils and other leucocytes, red blood cells, and platelets) [55,56], which requires NF-κB-mediated regulation [57]. In particular, the positioning of immune cells within the spleen and the ways in which their migration is orchestrated are both associated with its specific tailoring of the immune responses [58], and this hypothesis is well in agreement with our observations underlining the systemic impact of our NPs in DSS-induced colitis in mice. 

In view of the nanoparticle biodistribution, there was a distinct NP accumulation particularly in the intestine and appendix of diseased animals, compared to healthy mice. We expect that the DSS-induced injury of the colon increased the phagocytic potential of immune cells in various organs, with a high proportion of lymphoid organs (lung, intestine, appendix, etc.) followed by increased leucocyte degradation activity in the spleen thereafter (reduced accumulation compared to NP-treated healthy animals). 

Off-effects of NPs in the colon of healthy animals were very low. The increased COX-2 protein expression compared to non-treated healthy animals may be related to certain pro-inflammatory reactions, which seem to be very weak, since the anti-apoptotic protein Bcl-2 was increasingly expressed, together with the lowered expression of iNOS. The increased expression of IL-27 in the blood demonstrated the recognition of NPs in the body as foreign agents, since this mediator elicits an essential role of IL-27 signaling in regulating immune responses to extracellular protozoan infections [59,60]. 

## 5. Conclusions

In conclusion, our data show that our NPs carrying anti-p65 siRNA are able to alleviate colitis by suppressing the expression of several NF-κB-associated proteins. A clear down-regulating impact on NF-κB protein expression was early seen in colon leucocytes, whereas in whole colon tissue, a biased p65 regulation seems to take place as a result of the regenerative processes of the destroyed mucosa. There was also a systemic impact of the NP treatment in diseased mice, as seen by the modified blood mediator secretion, leucocyte load of the spleen, and mesenteric lymph nodes. The NPs revealed a good biocompatibility in healthy animals. Therefore, silica-coated calcium phosphate NPs carrying encapsulated siRNA against NF-κB p65 show promising features for the treatment of ulcerative colitis in the future. 

## Figures and Tables

**Table 1:** Score parameters for the assessment of colitis. Sum of scores represents the disease activity index (DAI).

Score	Weight Loss	Stool Consistency	Rectal Bleeding
0	No weight loss	Normal	No bleeding
1	1–5% weight loss	-	-
2	5–10% weight loss	Loose stool	Blood visible
3	10–15% weight loss	-	-
4	16–20% weight loss	Diarrhea	Strong rectal bleeding

**Table 2:** Characterization data of representative calcium phosphate nanoparticles from typical synthesis batches used in in vivo gene silencing experiments. CaP—calcium phosphate, DLS—dynamic light scattering, Dy—Dyomics, EU—endotoxin units, PDI—polydispersity index, PEI—polyethyleneimine, RNA—ribonucleic acid, SEM—scanning electron microscopy, siRNA—small interfering RNA. * Double-stranded siRNA molecules (duplexes).

	CaP/PEI-Dy734/siRNA/SiO_2_	CaP/PEI-Dy734/SiO_2_
Size by SEM/nm	80 ± 9	33 ± 2
Size by DLS/nm	253 ± 3	124 ± 1
PDI	0.18 ± 0.02	0.24 ± 0.01
Zeta potential/mV	22 ± 1	25 ± 3
Ca^2+^ concentration/µg mL^−1^	46	43
CaP concentration/µg mL^−1^	115	108
siRNA concentration/µg mL^−1^	36	-
siRNAs per nanoparticle *	11,896	-
PEI concentration/µg mL^−1^	240	240
Endotoxins/EU mL^−1^	0.04	0.07
Nanoparticle concentration/mL^−1^	3.94 × 10^10^	5.61 × 10^11^

## Data Availability

The raw/processed data required to reproduce these findings cannot be shared at this time due to technical or time limitations.

## Supplementary Materials

The following supporting information can be downloaded at: https://www.mdpi.com/article/10.3390/pharmaceutics14020419/s1, Table S1: Histo-morphological localization of protein expression in colon tissue after treatment with NPs, Figure S1: Impact of the NPs on blood composition during induction of DSS-mediated colitis in mice, Figure S2: Impact of NPs on clinical and histopathological features of the DSS model of colitis in mice at recovery day 1, Figure S3: Impact of NPs on the spleen of mice with DSS-mediated colitis at recovery day 1, Figure S4: Impact of NPs on p65 NF-κB and related proteins in mesenteric lymph nodes during induction of DSS-mediated colitis in mice at recovery day 1.
