# Supplementary material for: Nanoparticles Carrying NF-κB p65-Specific siRNA Alleviate Colitis in Mice by Attenuating NF-κB-Related Protein Expression and Pro-Inflammatory Cellular Mediator Secretion
Source: Pharmaceutics. 2022 Feb 15;14(2):419. doi: 10.3390/pharmaceutics14020419 (PMC8874689; doi:10.3390/pharmaceutics14020419)
Supplement: Supplementary file 1 [file pharmaceutics-14-00419-s001.zip › pharmaceutics-1580135-supplementary.pdf]

pharmaceutics

Supplementary Materials: Nanoparticles Carrying NF-κBp65-Specific siRNA Alleviate Colitis in Mice by Attenuating NF-κB-Related Protein Expression and Pro-Inflammatory Cellular Mediator Secretion

Elena K. Müller [1,†], Nataniel Białas [2,†], Matthias Epple [2,*] and Ingrid Hilger [1,*]

Supplementary Table S1. Histomorphological localization of protein expression in colon tissue after treatment with NPs. Since the expression pattern of selected protein was not different between recovery day 1 and 4, a summarized description is given.

Protein	NP treated	Colitis	Healthy
p65	Damaged goblet cells, inflammatory cells in mucosa, lymphoid organs, submucosa	Damaged goblet cells, inflammatory cells in mucosa, lymphoid organs, submucosa	Goblet cells, lymphoid organs, few leukocytes in lamina propria
IKB	Inflammatory cells (granulocytes, lymphocytes, macrophages)	Inflammatory cells (granulocytes, lymphocytes, macrophages)	mucus layer, few leukocytes in lamina propria
COX-2	Damaged mucosa, single cells in lymphoid organs	Damaged mucosa, single cells in lymphoid organs	Lamina propria
Bcl-2	Single lymphocytes in damaged mucosa,	Single lymphocytes in damaged mucosa,	Goblet cells (basal crypt regions), single lymphocytes in lamina propria and lymphoid organs
CD25, CD3, CD11c	Single cells in damaged mucosa and lymphoid organs	Single cells in damaged mucosa and lymphoid organs	Very few cells of lamina propria and lymphoid organs
F4/80	Throughout completely damaged mucosa and inflamed submucosa	Throughout completely damaged mucosa and inflamed submucosa	Few cells in lamina propria

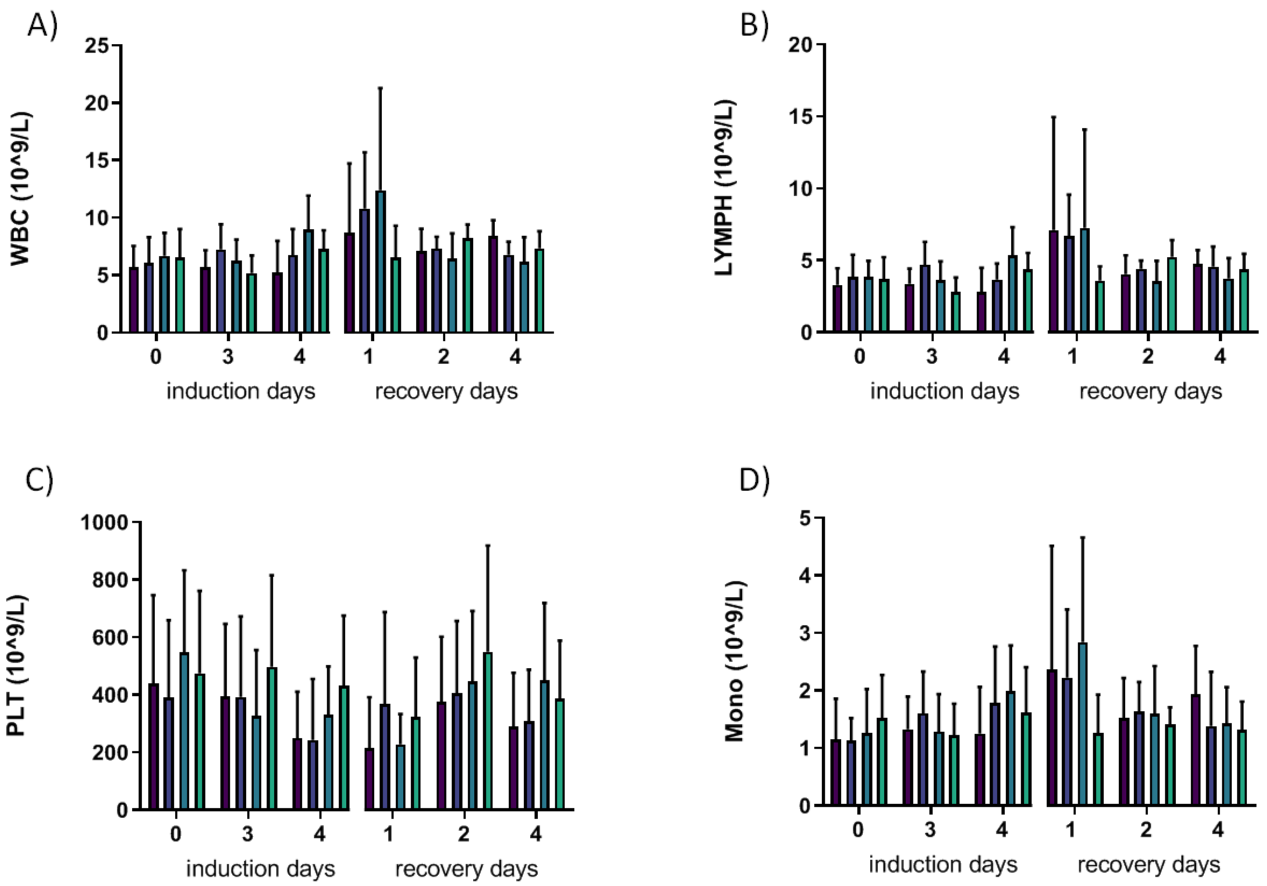

Supplementary Figure S1. Impact of the NPs on blood composition during induction of DSS-mediated colitis in mice. I) White blood cell count, II) lymphocyte count, III) platelet count, IV) Monocyte count. F: NPs in animals with colitis, C: Colitis only (no NPs), H: healthy animals (no NPs). Mean ± SD; *n* = 7 to 20.

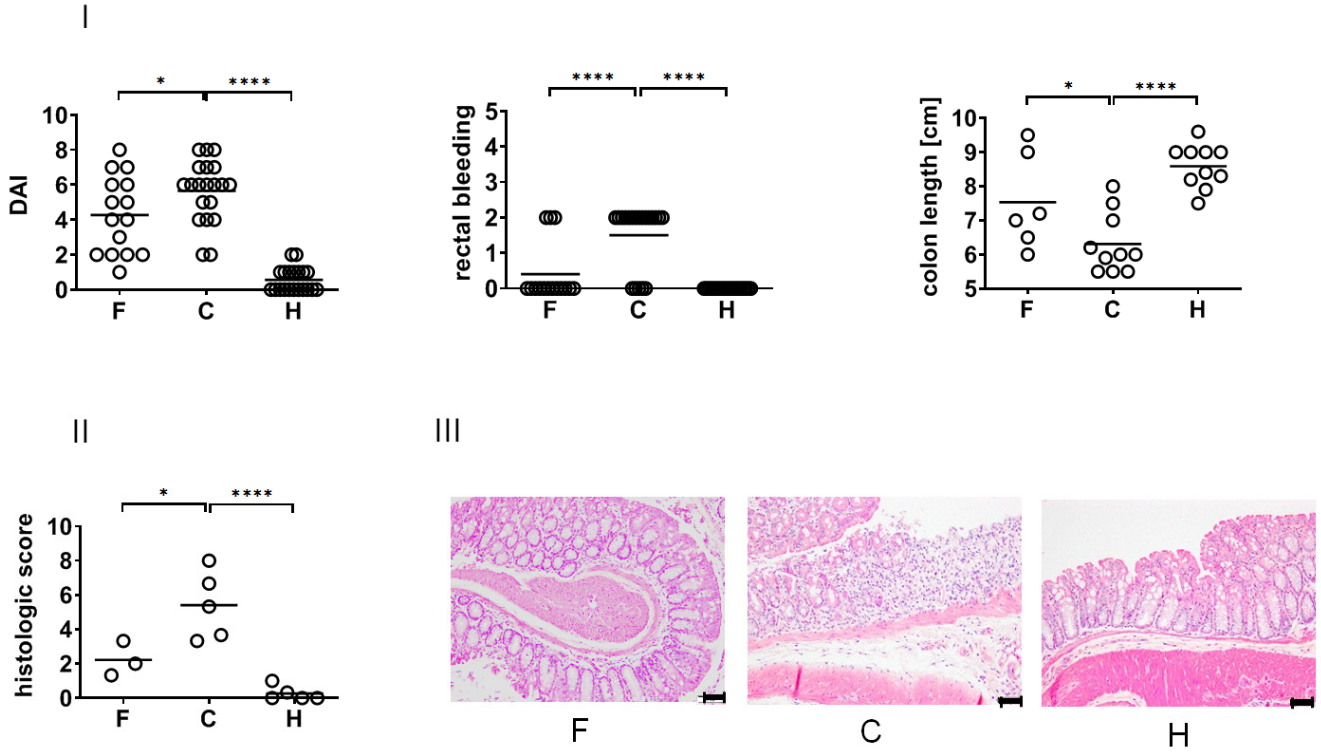

Supplementary Figure S2. **Impact of NPs on clinical and histopathological features of the DSS model of colitis in mice at the recovery day 1.** I) Clinical features, II) Histopathological scoring of damages in the mucosa and submucosa (see methods for scoring procedure) on the basis of HE stained histological slides from the colon. III) Representative microscopical pictures of the colon (HE, scale bars: 50 µm). F: NPs in animals with colitis, C: Colitis only (no NP), H: healthy animals (no NP). $n = 3$ to 20. Significant difference compared to group C with $p < 0.05$ (*), and $p < 0.0001$ (****).

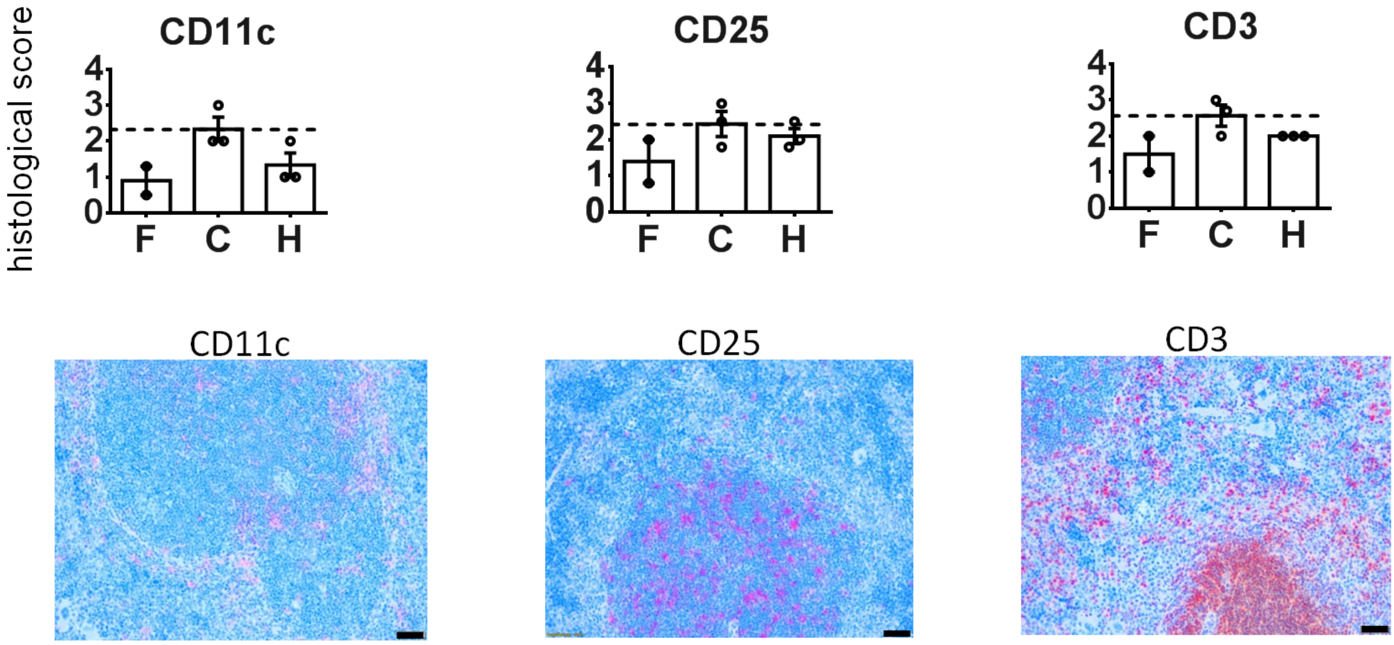

Supplementary Figure S3. Impact of NPs on in the spleen of mice with DSS-mediated colitis at recovery day 1. Number of CD11c, CD25, and CD3 in spleen tissue (histological scores, mean ± SD) and representative immuno-histological pictures of spleen from diseased animals (red: stained proteins, blue: hematoxylin counterstaining, scale bars: 200 µm). F: NPs in animals with colitis, C: Colitis only (no NP), H: healthy animals (no NPs). n = 2 to 5.

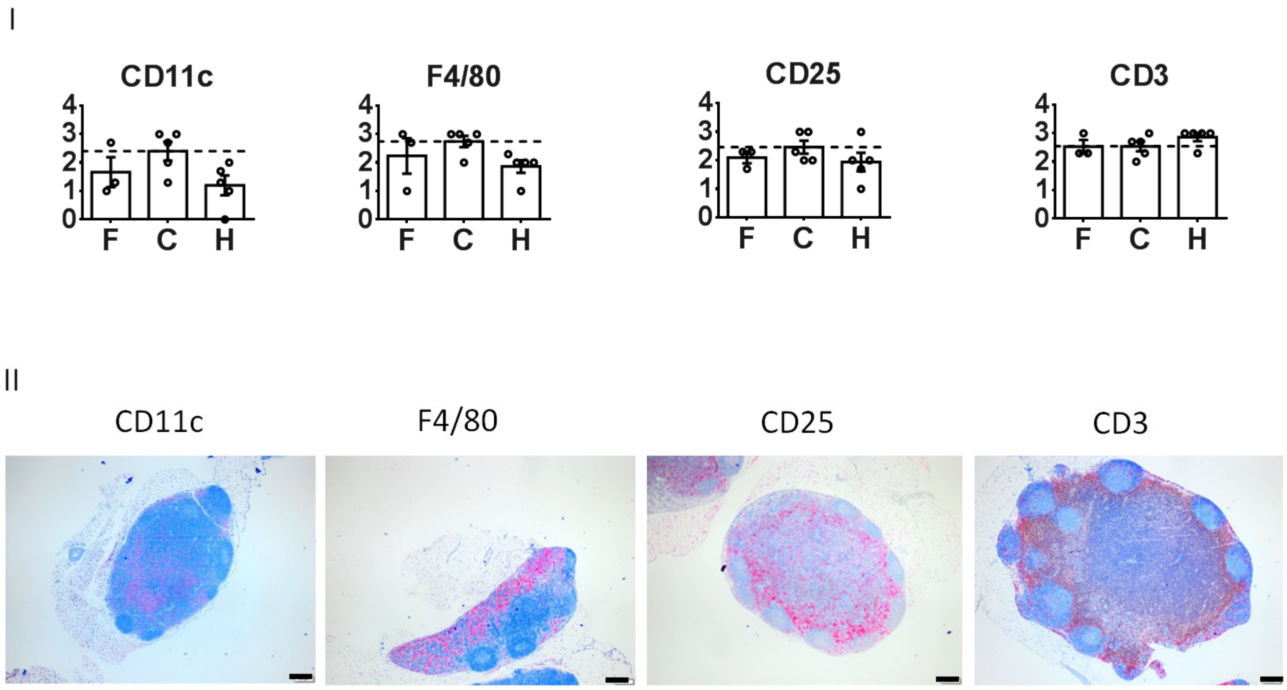

Supplementary Figure S4. Impact of NPs on p65 NF-κB and related proteins in mesenteric lymph nodes during induction of DSS-mediated colitis in mice at recovery day 1. I) Number of CD3, CD25, CD11c, II) Representative immune-histochemical sections of mesenteric lymph nodes of diseased mice (red: stained proteins, blue: hematoxylin counterstaining, scale bars: 200 µm). F: NPs in animals with colitis, C: Colitis only (no NPs), H: healthy animals (no NPs). Mean ± SEM. n = 3 to 5.
